# Work stress and burnout among radiology nurses: a cross-sectional study on the mediating role of effort-reward imbalance

**DOI:** 10.3389/fpubh.2025.1644328

**Published:** 2025-07-23

**Authors:** ChunQiao Wu, Qian Sun, Ping Liu, Jianbo Ni, Jianhua Gu

**Affiliations:** ^1^Department of Nursing, Sir Run Run Shaw Hospital, Zhejiang University School of Medicine, Hangzhou, China; ^2^Department of Radiology, Qilu Hospital of Shandong University (Qingdao), Qingdao, Shandong, China; ^3^Department of Medical Imaging, Affiliated Hospital of Jining Medical University, Jining, Shandong, China; ^4^Department of Emergency Medicine, Qilu Hospital of Shandong University, Jinan, Shandong, China

**Keywords:** occupational stress, effort-reward imbalance, burnout, radiology nursing, structural equation modeling

## Abstract

**Background:**

Radiology nurses face escalating occupational stressors associated with technological advancements and expanding clinical roles; however, evidence on burnout determinants in this specialized population remains limited. This study investigated the interplay between work stress, effort–reward imbalance (ERI), and burnout among radiology nurses, emphasizing the mediating role of ERI.

**Methods:**

This multi-center cross-sectional study enrolled 219 radiology nurses from six tertiary hospitals in China (January–March 2024). Validated instruments were used to assess work stress (Nurse Stressor Scale), ERI (Effort-Reward Imbalance Scale), and burnout (Maslach Burnout Inventory-General Survey). Structural equation modeling (SEM) was used to evaluate the mediation pathways, with covariates adjusted via multivariate logistic regression.

**Results:**

The participants presented elevated stress (59.22 ± 6.45), ERIs (mean ratio = 1.26 ± 0.82; 68.9% with ERI values >1), and near-clinical burnout levels (composite score = 3.17 ± 1.18). Emotional exhaustion (3.55 ± 1.95) was predominant. High stress (OR = 6.57, 95% CI = 3.58–12.04) and ERI (OR = 9.92, 95% CI=4.99–19.75) independently predicted moderate-to-severe burnout (38.8% prevalence). Nurses with prolonged weekly hours (65.85 ± 7.69 vs. 60.38 ± 6.22, *p* < 0.05) and chronic illness over time demonstrated heightened vulnerability. SEM revealed that ERI mediated 47.47% of the total effect of work stress on burnout (indirect effect = 0.047, 95% CI = 0.033–0.064), with distinct pathways through effort-reward disparity (31.31%) and overcommitment (17.17%).

**Conclusion:**

Chronic work stress and perceived effort–reward imbalance synergistically exacerbate burnout risk among radiology nurses, with the ERI mediating nearly half of the impact of stress. Targeted interventions addressing both technical demands and psychosocial inequities—particularly reward systems and workload equity—are urgently needed to mitigate occupational health crises in high-precision health care settings.

## Background

Burnout, characterized by emotional exhaustion, depersonalization, and diminished personal accomplishment, is a critical occupational hazard in health care (1). Recent epidemiological data reveal an alarmingly high prevalence of burnout among nurses in Chinese tertiary hospitals (2, 3), which poses substantial risks to clinical performance and patient safety (4). Chronic exposure to occupational stressors, such as excessive workload, staffing shortages, and emotionally demanding patient interactions, has been identified as a primary risk factor for nursing burnout (5). This persistent stress not only compromises individual psychological health but also threatens the quality and efficiency of health care services. Moreover, the effort–reward imbalance (ERI) model provides another crucial perspective for understanding occupational stress among nursing staff (6). The high-intensity efforts that nurses invest in prolonged standing, managing emergencies, and performing complex medical procedures often fail to receive commensurate economic compensation or career advancement opportunities. This long-term effort–reward imbalance is hypothesized to be linked to emotional exhaustion... and may be associated with more intense psychological stress responses, thereby being related to a higher risk of burnout. Based on the existing literature and the ERI model, we formulated the following hypotheses:

H1: Work stress is positively associated with burnout among radiology nurses.

H2: Effort-reward imbalance (ERI) is positively associated with burnout among radiology nurses.

H3: ERI may mediate the relationship between work stress and burnout, such that work stress is hypothesized to have an indirect effect on burnout through ERI.

Radiology nurses are a specialized group that faces significant work stress and occupational risk while executing high-precision technical tasks and managing radiation exposure. Despite their high-risk efforts, they frequently fail to receive corresponding economic and professional rewards, making the effort–reward imbalance particularly pronounced in this population. With the rapid advancement of radiological diagnostic and treatment technologies, the scope of radiology nursing continues to expand (7). In addition to traditional registration, appointment scheduling, and triage duties, radiology nurses must also assume responsibility for specialized examinations, emergency incidents, and communication coordination with patients, families, and other clinical departments (8). These diverse role requirements are associated with the work stress experienced by radiology nurses. Additionally, the growing demand for imaging services has increased the workload of radiology nurses, as relative staffing shortages lead to frequent overtime and multiple role transitions, further heightening the risk of burnout.

While previous studies have explored the relationships among work stress, effort-reward imbalance, and burnout (9–12), relatively few studies have focused on specific populations of radiology nurses. This study aims to investigate the relationships among work stress, effort-reward imbalance, and burnout among radiology nurses, with a particular emphasis on the mediating role of effort-reward imbalance between work stress and burnout, providing theoretical foundations and empirical support for the development of effective interventions.

## Methods

### Study participants

This study employed a cross-sectional design. Between January and March 2024, data were collected using convenience sampling from eligible radiology nursing staff at Qilu Hospital of Shandong University (Qingdao), the Affiliated Hospital of Jining Medical University, the First Affiliated Hospital of Zhengzhou University, West China Hospital of Sichuan University, the First Affiliated Hospital of Chongqing University, and Peking University First Hospital. The inclusion criteria were as follows: (1) current employment as a radiology nurse, (2) work experience ≥1 year, (3) possession of a nursing license, and (4) informed consent and voluntary participation. The exclusion criteria included rotating, visiting, or nurses on leave.

This study incorporated 20 observed variables: 9 demographic baseline variables, 5 dimensions from the Nurse Stressor Scale, 3 dimensions from the Effort-Reward Imbalance Scale, and 3 dimensions from the Burnout Inventory. Following the sample size determination principle (5–10 times the expected number of variables with an additional 10% allowance for potential attrition), we aimed to recruit ≥210 participants, meeting the minimum sample requirement for AMOS structural equation modeling analysis (200 cases). The final sample included 219 radiology nursing professionals, which exceeded the predetermined sample size standard. This study received approval from the Ethics Committee of Qilu Hospital of Shandong University, Qingdao Campus (KYLL-KS-2024250).

### Survey instruments

(1) General Information Questionnaire: This questionnaire was developed by the research team and included basic demographic information such as age, gender, educational background, professional title, and years of work experience. It also included items on weekly work hours, overtime, shift work patterns, and self-reported chronic disease status (defined as having a physician-diagnosed condition such as hypertension, diabetes, or thyroid disorders requiring ongoing management).

(2) Nurse Stressor Scale: We utilized the Chinese version of the Nurse Stressor Scale (CNSS), originally modified and validated by Li and Liu (13) to accommodate the cultural and professional characteristics of health care providers in China. This validated instrument contains 35 items across five dimensions: (1) the nursing profession and work demands, (2) time allocation and workload, (3) the work environment and resource availability, (4) patient care challenges, and (5) management and interpersonal relationships. The items were rated on a 4-point Likert scale ranging from 1 (no stress) to 4 (extreme stress), with higher composite scores (theoretical range: 35–140) indicating greater perceived stress. Participants scoring above the scale's mean value (calculated across all items) were categorized as experiencing high stress levels. The scale demonstrated good internal consistency in our sample (Cronbach's α = 0.821).

(3) ERI Scale: Effort-Reward Imbalance (ERI) Scale: We administered the Chinese version of the ERI scale transculturally adapted through rigorous forwards-backwards translation procedures and validated Li et al. (14), building upon the original framework established by Siegrist. This validated instrument consists of three subscales: (1) effort (6 items), (2) reward (11 items), and (3) overcommitment (6 items). The effort and reward subscales employ a 5-point Likert scale (1 = minimally applicable, 5 = strongly applicable). The ERI ratio was calculated with the following formula: (Σ Effort items)/(Σ Reward items) × 0.545, with values >1.0 indicating significant effort-reward imbalance. The overcommitment subscale uses a 4-point Likert scale (total score range: 6–24), where participants scoring in the upper tertile of the sample distribution are classified as having elevated overcommitment. This scale demonstrated excellent reliability in our cohort, with an overall Cronbach's α of 0.880.

(4) Maslach Burnout Inventory-General Survey (MBI-GS): we implemented the transculturally adapted Chinese version validated by Li and Shi (15), which preserves the original three-factor structure. This version was developed in collaboration with Michael Leiter, one of the original MBI authors, and has established strong psychometric properties for use in the Chinese context. This 15-item instrument assesses burnout through three subscales: emotional exhaustion (5 items), depersonalization (4 items), and personal accomplishment (6 items, reverse-coded), using a 7-point frequency scale (0 = never, 6 = daily). Following the scoring protocol established by Li and Shi (15) for the Chinese context, composite burnout scores were computed using their validated weighted formula: (0.4 × mean emotional exhaustion score) + (0.3 × mean depersonalization score) + (0.3 × [6—mean personal accomplishment score]). This weighting is based on the factor loadings from the original Chinese validation study, where emotional exhaustion carries the greatest weight, reflecting its central role in the burnout construct. The formula provides a single index of burnout severity, which is particularly suitable for our structural equation modeling. Scores ≥3.5 on this composite scale are considered indicative of moderate-to-severe burnout, consistent with established norms for this version of the instrument. Excellent internal consistency was observed in our sample (α = 0.825).

### Data collection

This study employed a commercial survey platform (www.wjx.cn) for electronic questionnaire development and distribution. To ensure methodological rigor, we conducted pilot testing with 22 radiology nurses at the Affiliated Hospital of Jining Medical University, verifying instrument feasibility, technical stability, and average completion time (15–20 min).

Before the main data collection, the research team conducted standardized training sessions with charge nurses from the participating hospitals, detailing the study objectives, eligibility criteria (inclusion: active clinical nurses; exclusion: administrative/educational staff), and completion protocols. This cascading training model ensured consistent implementation across sites while maintaining participant anonymity.

The final survey was distributed through WeChat groups with a unique QR code with embedded device fingerprinting technology. To enhance data quality, (1) required-response formatting prevented item nonresponse; (2) geographic IP filtering restricted participation to authorized hospitals; and (3) cryptographic device identification limited submissions to one per mobile device.

### Data quality control

Among the 243 distributed questionnaires, 219 were returned (response rate = 90.12%). A two-stage validation process was implemented: (1) automated screening eliminated records with completion times <300 seconds (indicating random response); (2) manual review by two independent raters removed questionnaires showing response patterns (e.g., straight-lining) or logical inconsistencies (e.g., conflicting demographic/work-hour reports). All 219 retained questionnaires met stringent validity criteria. Given the cross-sectional, self-report nature of the data, several measures were taken to control for potential common method variance. Procedurally, we ensured participant anonymity and confidentiality to reduce social desirability bias. The use of multiple, well-validated instruments with varying Likert scale formats (e.g., 4-point, 5-point, and 7-point scales) also helped to mitigate uniform response patterns. Statistically, we performed Harman's single-factor test by entering all items from the primary study scales (NSS, ERI, MBI-GS) into an unrotated exploratory factor analysis. The results indicated that the first factor accounted for 31.6% of the total variance, which is below the conventional 40% threshold, suggesting that common-method variance was not a major concern in this study.

### Statistical analysis

All analyses were conducted using R 4.3.1 with the lavaan package for structural equation modeling (SEM). Continuous variables with a normal distribution (Shapiro–Wilk test *p* > 0.05) were reported as means ± standard deviations (SDs), nonnormal variables are reported as medians [IQRs], and categorical variables are reported as counts (%). Bivariate correlations among work stress (NSS total score), effort-reward imbalance (ERI ratio), and burnout (MBI-GS composite) were examined using Pearson's r test with Bonferroni correction for multiple comparisons.

Binary logistic regression was used to model moderate-to-severe burnout (dichotomized at ≥3.5) as an outcome variable, incorporating work stress (NSS mean score) and ERI status (imbalance: ERI > 1) as primary predictors. Model assumptions were verified through variance inflation factors (<2.0) and the Hosmer–Lemeshow goodness-of-fit test (*p* = 0.62). The results are reported as odds ratios (aORs) with 95% confidence intervals (CIs), controlling for age, shift work, and clinical experience as covariates.

SEM analysis in AMOS 28.0 was used to evaluate a hypothesized parallel mediation model in which work stress was associated with burnout via effort-reward imbalance and overcommitment, using maximum likelihood estimation with Bollen-Stine bootstrap correction (2,000 resamples). Model fit was assessed via the comparative fit index (CFI > 0.95), root mean square error of approximation (RMSEA < 0.06), and standardized root mean squared residual (SRMR < 0.08). Mediation effects were considered significant if 95% bias-corrected bootstrap CIs excluded zero. All tests were two-tailed with α = 0.05.

### Sensitivity analyses

To assess the robustness of our findings, two sensitivity analyses were conducted. First, to ensure our results were not solely driven by participants with extreme work hours, we examined the core bivariate correlations among work stress, ERI, and burnout within the subsample of nurses working ≤ 60 h/week (*n* = 89). Second, to test the influence of potential outliers, we screened key continuous predictor variables (e.g., ERI ratio, NSS score, and ERI subscales) for values exceeding 3 standard deviations from the mean. The screening confirmed that the core associations persisted in the non-extreme work-hour group and identified only a negligible number of statistical outliers (e.g., a single outlier for the ERI ratio in the entire sample). Given these results, the original analyses were deemed robust and not unduly influenced by specific subgroups or extreme values.

## Results

### Scores for stresses, effort-reward imbalance, and burnout among radiology nurses

The descriptive statistics for the main study variables are presented in Table 1. The mean total score on the CNSS for the sample was 59.22 ± 6.45. Among its subscales, the ‘Management and Interpersonal Relationships' dimension yielded the highest score (12.32 ± 3.24). For the ERI scale, the mean ERI ratio was 1.26 ± 0.82, with 68.9% of participants having a ratio >1. The mean overcommitment score was 15.22 ± 5.15. For the MBI-GS, the mean composite burnout score was 3.17 ± 1.18. Within the three burnout dimensions, emotional exhaustion had the highest mean score (3.55 ± 1.95).

**Table 1:** General information and factors influencing occupational burnout among radiology nurses.

**Scale and dimensions**	**Items**	**Score (mean ±SD)**
**Nurse Stressor Scale**		
Nursing profession and work demands	7	11.58 ± 3.04
Time allocation and workload	7	11.68 ± 2.82
Work environment and resources	7	11.62 ± 3.15
Patient care challenges	7	12.02 ± 2.94
Management and interpersonal relationships	7	12.32 ± 3.24
Total score		59.22 ± 6.45
**Effort-Reward Imbalance Scale**		
Effort	6	18.83 ± 3.47
Reward	11	33.47 ± 12.96
ERI ratio		1.26 ± 0.82
Overcommitment	6	15.22 ± 5.15
**Burnout Inventory**		
Emotional exhaustion	5	3.55 ± 1.95
Depersonalization	4	3.16 ± 2.04
Reduced personal accomplishment	6	3.33 ± 1.73
Composite score		3.17 ± 1.18

**Figure 1:**
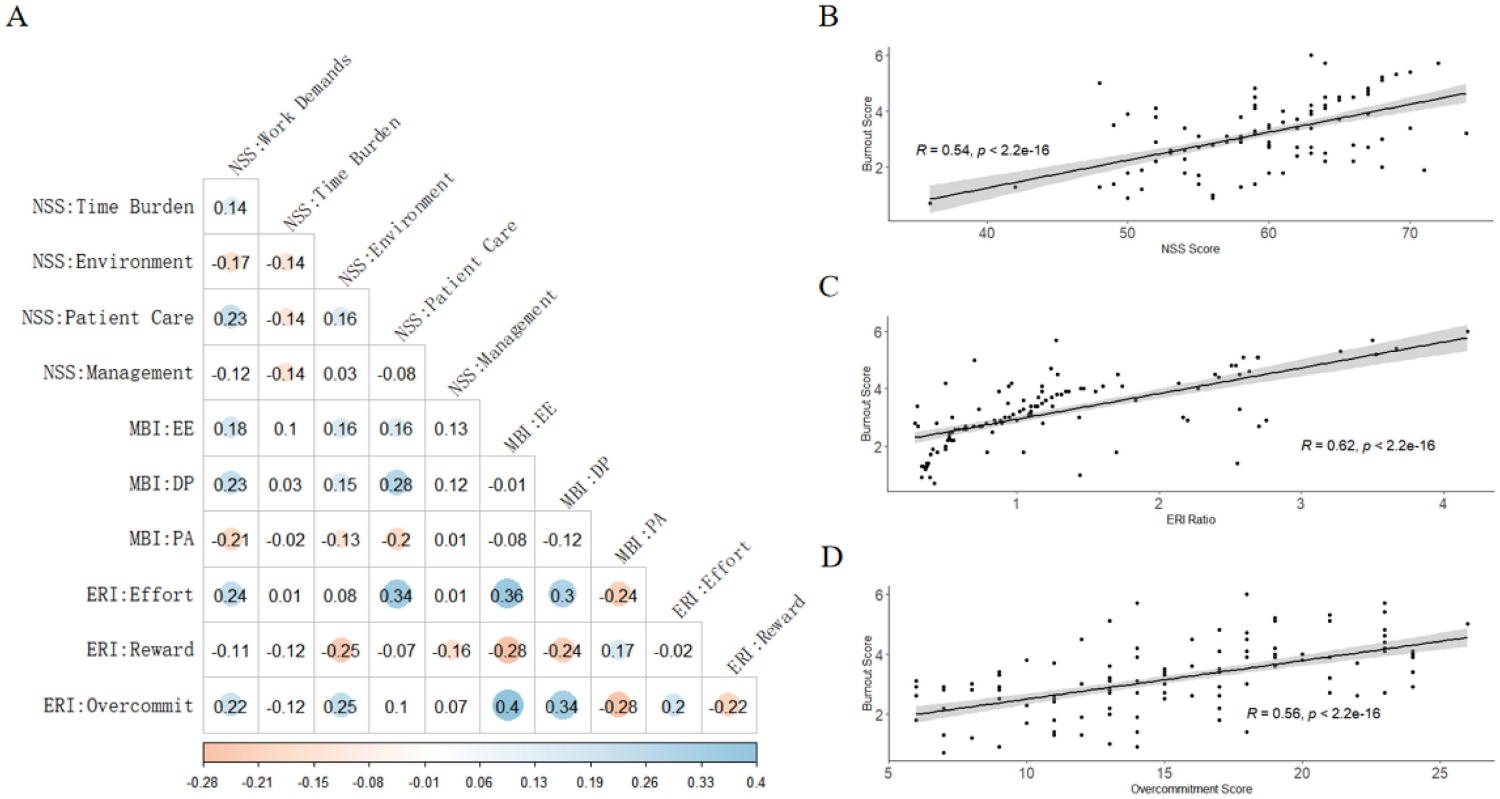
Correlation analysis of work stress, effort-reward imbalance, and burnout among radiology nurses. **(A)** A heatmap illustrating the correlations between the dimensions of the studied variables. Blue shading indicates a positive correlation, while orange indicates a negative correlation (*P* < 0.05). The absence of color indicates a non-significant correlation (*P* > 0.05). **(B)** Scatter plot showing the positive correlation between work stress (NSS total score) and burnout (MBI-GS composite score). **(C)** Scatter plot showing the positive correlation between effort-reward imbalance (ERI ratio) and burnout. **(D)** Scatter plot showing the positive correlation between work stress and effort-reward imbalance. NSS, Nurse Stressor Scale (with domains: Work Demands, Time Burden, Environment, Patient Care, Management); MBI, Maslach Burnout Inventory (with subscales: EE, Emotional Exhaustion; DP, Depersonalization; PA, Personal Accomplishment); ERI, Effort-Reward Imbalance model (with components: Effort, Reward, and Overcommitment).

Significant intercorrelations emerged among work stress, ERI, and burnout (Figure 1A). Pearson analysis revealed that burnout was positively correlated with work stress (r = 0.54, *P* < 0.05; Figure 1B) and the ERI (r = 0.62, *P* < 0.05; Figure 1C), whereas work stress was moderately associated with the ERI (r = 0.44, *P* < 0.05; Figure 1D).

### Comparison of characteristics between radiology nurses with different levels of burnout

Among the 219 nurses, 85 (38.8%) experienced moderate-to-severe burnout (MBI-GS score ≥3.5). Compared with their non-burnout counterparts (all *P* < 0.05), this group was significantly older (32.13 ± 4.74 vs. 29.41 ± 5.02 years), had longer weekly working hours (65.85 ± 7.69 vs. 60.38 ± 6.22 h), and reported a higher prevalence of chronic disease (9.4% vs. 1.5%; p < 0.05), had extended over time (≥10 h/week: 57.6% vs. 34.3%), and had more radiology experience (>10 years: 25.9% vs. 17.2%). No significant group differences existed in terms of marital status, education, or family caregiving responsibilities (*P* > 0.05) (Table 2).

**Table 2:** Comparison of general condition and influencing factors of occupational burnout among radiology nurses.

**Characteristics**	**Non-burnout**	**Moderate-severe burnout**	***P***
	***n*** = **134**, ***n*** **(%)**	***n*** = **85**, ***n*** **(%)**	
Age (years)	29.41 ± 5.02	32.13 ± 4.74	0.001
<25	23 (17.2)	1 (1.2)	
25–30	57 (42.5)	37 (43.5)	
>30	54 (40.3)	47 (55.3)	
Professional title			0.006
Staff nurse	41 (30.6)	18 (21.2)	
Junior nurse	46 (34.3)	39 (45.9)	
Senior nurse	45 (33.6)	20 (23.5)	
Deputy chief/chief nurse	2 (1.5)	8 (9.4)	
Marital status			0.152
Married	94 (70.1)	59 (69.4)	
Unmarried	39 (29.1)	22 (25.9)	
Divorced	1 (0.7)	4 (4.7)	
Weekly working hours	60.38 ± 6.22	65.85 ± 7.69	<0.001
Overtime frequency			<0.001
Rare/ < 5 hours weekly	45 (33.6)	10 (11.8)	
5–10 hours weekly	43 (32.1)	26 (30.6)	
>10 hours weekly	46 (34.3)	49 (57.6)	
Education level			0.068
Associate degree	49 (36.6)	28 (32.9)	0.068
Bachelor's degree	70 (52.2)	49 (57.6)	
Master's degree or higher	15 (11.2)	8 (9.4)	
Radiology experience (years)			0.04
>10	23 (17.2)	22 (25.9)	
5–10	24 (17.9)	6 (7.1)	
≤5	87 (64.9)	57 (67.1)	
Elder care stress			0.247
No	108 (80.6)	62 (72.9)	
Yes	26 (19.4)	23 (27.1)	
Childcare stress			0.264
No	62 (46.3)	32 (37.6)	
Yes	72 (53.7)	53 (62.4)	
Chronic disease			0.016
No	132 (98.5)	77 (90.6)	
Yes	2 (1.5)	8 (9.4)	

### Impact of work stress and effort–reward imbalance on burnout

Compared with the reference groups, the high-stress, effort-reward imbalance (ERI > 1), and overcommitment groups presented significantly greater burnout prevalence and severity (all *P* < 0.05, Table 3). Univariate logistic regression (Model 1) revealed strong associations: high stress (OR = 6.57, 95% CI = 3.58–12.04), ERI (OR = 9.92, 95% CI = 4.99–19.75), and overcommitment (OR = 15.22, 95% CI = 7.18–32.29) were significantly associated with a higher likelihood of burnout. These relationships persisted in multivariate analysis (Model 2) after adjusting for baseline covariates, with adjusted ORs maintaining statistical significance (*P* < 0.05 for all predictors).

**Table 3:** Associations of work stress and effort-reward imbalance on occupational burnout.

**Scale**	**Burnout status**		**Model 1^a^**		**Model 2^b^**	
	**Non-burnout**	**Moderate-severe burnout**	**OR**	**95%CI**	**OR**	**95%CI**
Stress level	57.46 ± 6.16	61.99 ± 5.94				
Low stress	95	23	Ref		Ref	
High stress	39	62	6.57	3.58–12.04	5.05	2.29–11.14
ERI Status	0.96 ± 0.65	1.73 ± 0.83				
Balanced (ERI ≤ 1)	86	13	Ref		Ref	
Imbalanced (ERI >1)	48	72	9.92	4.99–19.75	24.71	8.16–74.89
Overcommitment	12.81 ± 4.39	19.02 ± 3.79				
No	123	36	Ref		Ref	
Yes	11	49	15.22	7.18–32.29	15.39	5.76–41.08

a Univariate logistic regression analysis.

b Multivariable logistic regression analysis adjusted for all baseline covariates listed in Table 1.

### Analysis of potential mediation by effort-reward imbalance in the association between work stress and burnout

**Figure 2:**
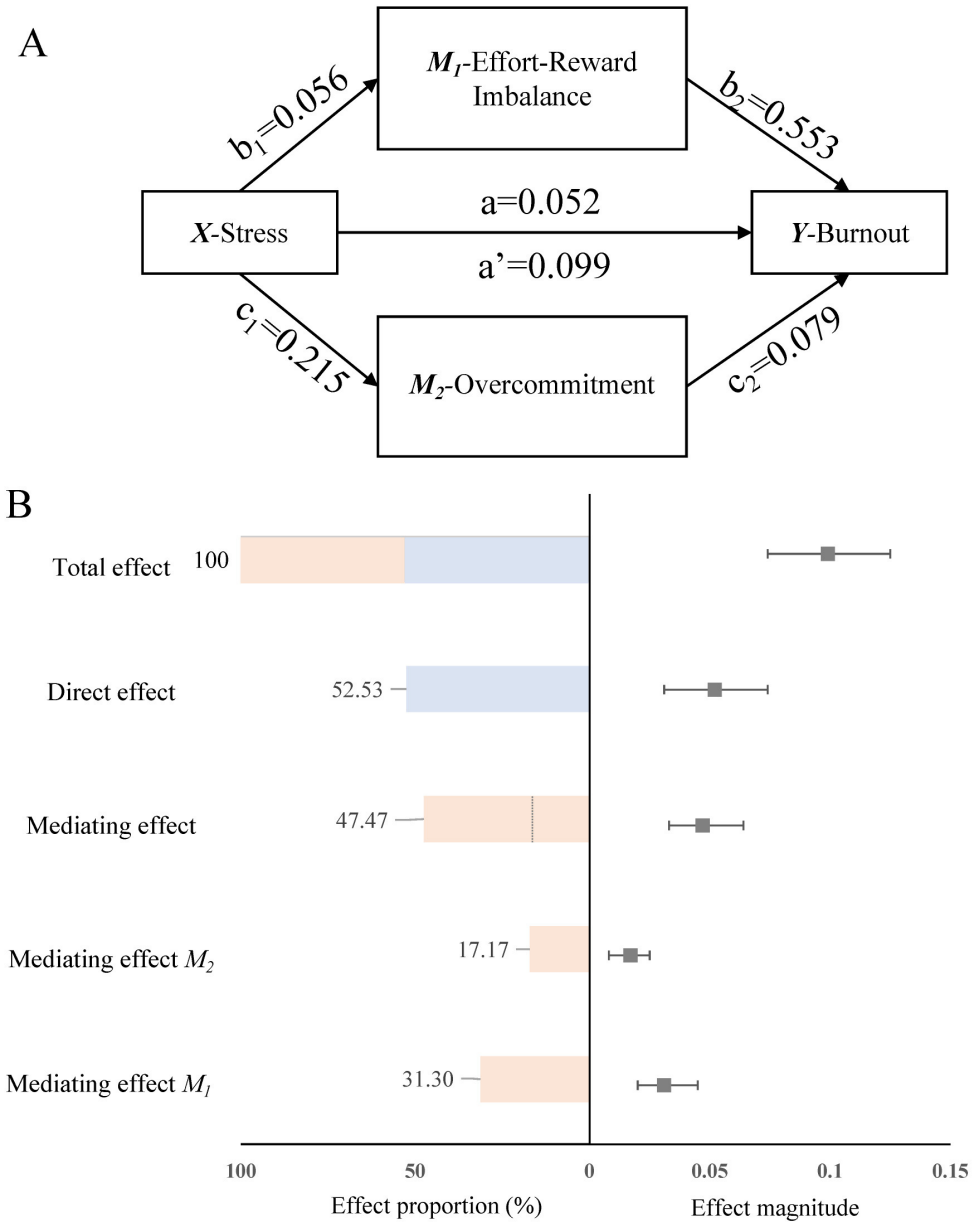
Mediation analysis of work stress, effort-reward imbalance, and occupational burnout among radiology nurses. **(A)** The structural equation model illustrates the direct and indirect pathways from work stress to burnout. Standardized path coefficients are shown. Solid arrows indicate significant paths (*p* < 0.05). **(B)** A forest plot and bar chart decomposing the total effect into its constituent direct and indirect (mediated) components, with corresponding 95% confidence intervals and percentage contributions. Path definitions: *a'* = total effect of work stress on burnout; *a* = direct effect of work stress on burnout; *b1* = effect of work stress on ERI; *b*_2_ = effect of ERI on burnout; *c1* = effect of work stress on overcommitment; *c*_2_ = effect of overcommitment on burnout. The indirect effect via ERI is calculated as *b1* × *b*_2_, and the indirect effect via overcommitment is *c1* × *c*_2_.

The SEM examining the mediating role of ERI and overcommitment is presented in Figure 2. As shown in Figure 2A, a significant total effect was observed in the association between work stress and burnout (path *a'* = 0.099, 95% CI: 0.074–0.125). This total effect could be partitioned into a significant direct effect and two significant indirect effects. The direct effect of work stress on burnout (path *a*) was 0.052 (95% CI: 0.031–0.074), accounting for 52.53% of the total effect. The indirect effects, which collectively mediated 47.47% of the relationship, were channeled through two pathways. The first pathway, representing the potential indirect effect via ERI, showed a statistically significant mediating effect (indirect effect = 0.031, 95% CI: 0.020–0.045), explaining 31.31% of the total effect. This was composed of the path from work stress to ERI (path *b1* = 0.056) and the path from ERI to burnout (path *b*_2_ = 0.553). The second pathway, “work stress → overcommitment → burnout,” also represented a significant potential indirect effect (indirect effect = 0.017, 95% CI: 0.008–0.025), contributing 17.17% to the total effect. This pathway consisted of the path from work stress on overcommitment (path *c1* = 0.215) and the subsequent effect of overcommitment on burnout (path *c*_2_ = 0.079). Figure 2B visually decomposes these effects, illustrating the relative contribution of the direct and mediated pathways.

## Discussion

This study provides a comprehensive examination of the interrelationships among work stress, effort–reward imbalance, and burnout among radiology nurses, offering vital insights into their occupational health risks. Our study revealed a burnout prevalence rate of 38.8%, with emotional exhaustion identified as the most common symptom. Work-related stress and effort-reward imbalance were strongly positively correlated with burnout (r = 0.54 and r = 0.62, respectively). Multivariate analysis further established these factors as independent risk contributors, even after adjusting for demographic and occupational variables. Nurses with extended professional tenure, longer weekly working hours (65.85 ± 7.69 compared with 60.38 ± 6.22), and frequent overtime were particularly susceptible, indicating the cumulative association of prolonged exposure to occupational stressors. Notably, the effort–reward imbalance was identified as a significant potential mediator in the relationship between work stress and burnout, accounting for nearly half (47.47%) of this association. This finding underscores that the psychological associations of work stressors appear stronger when nurses perceive a disparity between their substantial efforts and the rewards, recognition, or advancement opportunities they receive. Consequently, these results underscore the critical importance of addressing both the technical demands of radiology nursing and the associated psychosocial factors.

Burnout is a pressing occupational health concern for nursing professionals, impacting their physical and mental wellbeing, diminishing care quality, contributing to medical disputes, and increasing turnover intentions, thus warranting significant attention. With rapid advancements in imaging technology, the scope of radiology nursing has broadened, and nurses' roles have become increasingly multifaceted (16). In addition to routine tasks such as registration, scheduling, and triage, radiology nurses now manage specialized examinations, respond to emergencies, communicate with patients and families, and coordinate with other departments. This heightened workload and stress render them particularly susceptible to burnout (17). Our observed 38.8% prevalence of moderate-to-severe burnout appears substantially higher than the 11.23% global pooled prevalence reported in a multinational meta-analysis (18). However, this comparison must be interpreted with caution may be related to significant methodological differences. The meta-analysis included studies using various burnout instruments and diagnostic cut-offs, whereas our study employed the Chinese-validated MBI-GS with a specific composite score formula (≥3.5) to define moderate-to-severe burnout. This methodological heterogeneity can heavily influence reported prevalence rates. Nonetheless, our finding aligns with and even exceeds the rates reported in other high-risk nursing specialties within China. For example, a meta-analysis of Chinese mental health nurses revealed a 28.1% prevalence of high emotional exhaustion (19). This disparity may be partially explained by the compounding pressures of radiation safety protocols, rapid technological adaptation, and prolonged overtime, which are particularly pronounced in radiology settings. Notably, senior nurses with extended tenure emerged as a high-risk subgroup, illustrating how clinical expertise becomes a vulnerability under chronic reward deprivation and unmet career advancement expectations.

Our findings identify older age, prolonged working hours, and frequent overtime as critical burnout risk factors among radiology nurses, corroborating broader patterns observed in nursing populations. The older nurses in our cohort face compounded stressors: they typically assume managerial and educational roles while confronting career stagnation due to limited promotion opportunities, which erodes professional identity—a known protective factor against burnout (20). This aligns with studies showing that nurses aged 40–45 years' experience peak burnout levels, often stemming from work-family conflicts and unresolved systemic pressures (21). Frequent overtime, a proxy for excessive work investment, exacerbates physical and emotional depletion, creating a cyclical relationship between chronic stress and diminished job engagement (22). Notably, while younger nurses globally report higher burnout rates, our findings highlight that older radiology nurses—despite their clinical expertise—remain disproportionately vulnerable due to distinct demands of their specialty such as radiation safety compliance and rapid technological adaptation.

A particularly noteworthy finding is the high average of weekly work hours reported by the nurses (mean > 60 h). It is crucial to interpret this figure in the context of how it was measured. Our survey instrument prompted participants to report their “actual total weekly work time,” explicitly instructing them to include not only regular shifts and overtime but also “on-call duties, any on-site time, standby, and administrative tasks.” This comprehensive measurement explains why the reported hours appear to exceed standard national guidelines for scheduled work. It suggests that a substantial portion of a radiology nurse's workload consists of activities beyond formal shift boundaries—a form of “hidden work” that is often uncompensated but contributes significantly to physical and mental load. The implications are clear: the true occupational burden on these nurses is far greater than what is captured by conventional scheduling metrics. This sustained high workload, encompassing both direct patient care and peripheral duties, is a primary driver of exhaustion and burnout. Therefore, our data highlight an urgent need for healthcare organizations to look beyond formal schedules and develop strategies that address the total workload. This could involve optimizing staffing models to account for these informal duties, streamlining administrative workflows, and fostering a culture that protects nurses' off-duty time.

Correlation analyses confirmed positive associations between burnout and both work stress and effort-reward imbalance, with logistic regression further confirming that high-stress, effort-reward imbalance, and overcommitment groups faced significantly elevated burnout risks, even after adjusting for baseline factors. These findings corroborate prior studies identifying stress and ERI as critical burnout risk factors (23). Shah et al. noted that high work demands paired with low control heighten burnout risk, whereas the ERI model posits that an imbalance between effort and reward is a primary correlate (24). When substantial effort yields inadequate material rewards, promotion, or recognition, frustration and exhaustion ensue. Radiology nurses operate in a high-intensity, high-stress environment driven by increased examination volumes due to technological advancements and rising demands for quality amid heightened patient safety expectations. These pressures compel nurses to undertake more tasks with greater responsibility within constrained timeframes. However, imperfect performance evaluation and promotion systems in many hospitals fail to adequately reward their efforts (25), leading to a persistent high-pressure environment and effort-reward imbalance that precipitates burnout. To address this, administrators should optimize radiology nursing staffing on the basis of workload and service demands; refine evaluation and promotion systems to reflect effort, skills, and quality; and provide commensurate rewards and advancement opportunities. Nurses, in turn, should enhance self-regulation, foster a positive work outlook, prioritize self-care, and seek psychological support when needed to better manage occupational stress.

This study is among the first to explore ERI as a potential mediator in the association between work stress and burnout among radiology nurses. Our structural equation model is consistent with a pathway in which work stress is indirectly associated with burnout via ERI and overcommitment, with mediating effects accounting for 47.47% of the total effect. This suggests a potential mechanism by which intense work stress is linked to higher levels of effort–reward imbalance and overcommitment, which in turn are associated with increased burnout. Sources of stress for radiology nurses include continuous learning demands from technological updates, accelerated work pace due to rising examination volumes, and escalating service quality expectations (26). These factors necessitate complex tasks—such as supporting specialized examinations and handling emergencies—within limited time, fostering physical and mental exhaustion and workplace dissatisfaction. In high-stress settings, nurses often perceive their efforts as inadequately rewarded in terms of salary, advancement, or satisfaction, leading to frustration and disengagement that exacerbates burnout (27). While technological innovation holds long-term promise, its immediate implementation can introduce new challenges. This continuum—from stress to effort-reward imbalance to burnout—underscores the intricate interplay of the work environment, psychological states, and occupational health. Interventions should thus alleviate stress while aligning effort and reward to enhance professional fulfillment and prevent burnout at its root.

It is important to reconcile these mediation results with the findings from our logistic regression analysis (Table 3). In the regression model, ERI demonstrated a much larger odds ratio (OR = 24.71) for predicting burnout than work stress (OR = 5.05). These two results, while seemingly different, are in fact complementary and reflect the different nature of prediction vs. explanation. The logistic regression highlights that ERI, as a proximal psychosocial state, is an exceptionally strong predictor of burnout. The SEM, in contrast, provides a mechanistic explanation, showing that a substantial portion of the effect from the more distal work stress is channeled through ERI. Taken together, these findings paint a comprehensive picture: work stress creates the conditions for an effort-reward imbalance, and this state of imbalance is a powerful, direct precursor to experiencing burnout. This dual evidence strongly validates the targeting of ERI in future interventions.

While this study has several limitations, the robustness of our primary findings was supported by sensitivity analyses that accounted for extreme work hours and statistical outliers. First, its cross-sectional design precludes establishing causality among work stress, effort–reward imbalance, and burnout; longitudinal studies are needed to confirm temporal relationships. Second, our sampling strategy introduces potential selection bias. The use of convenience sampling from six major tertiary hospitals may limit the generalizability of our findings to secondary hospitals or different regions. More critically, our exclusion of rotating and visiting nurses may have led to an underestimation of the true burnout prevalence. As temporary staff often face greater workload instability and are less integrated into departmental reward systems, they may experience even higher levels of stress and burnout. Consequently, our reported burnout prevalence of 38.8% should be interpreted as a potentially conservative estimate for the entire radiological nursing workforce. Future research employing a more inclusive, stratified sampling design is crucial to capture the unique occupational health risks faced by these transient staff members. Third, reliance on self-reported measures introduces potential response bias or social desirability effects; incorporating objective data or multisource assessments could strengthen findings. Fourth, some of our subgroup analyses were based on small sample sizes, limiting their statistical power. For instance, the finding that nurses with chronic diseases reported higher burnout was based on a small number of participants (n = 10 in total), which restricts the generalizability of this specific result. Fifth, our choice of measurement instruments warrants discussion. We used the MBI-General Survey (MBI-GS) instead of the MBI-Human Services Survey (MBI-HSS), which is often preferred for healthcare settings. Our decision was guided by the availability of a robustly validated Chinese MBI-GS version with a composite score formula well-suited for our SEM analysis. Nevertheless, we acknowledge the MBI-HSS might have captured more patient-centered aspects of burnout. Additionally, while we dichotomized burnout for the logistic regression analysis, we recognize that current psychometric consensus increasingly favors treating burnout as a continuous spectrum. Our primary SEM analysis, however, did use the continuous composite score, thereby retaining the full variance of the data for the main hypothesis testing. Finally, our analysis did not stratify by gender, which may obscure important differences in how work stress and ERI affect burnout among male and female radiology nurses. This is a notable limitation, as emerging evidence suggests gender plays a critical moderating role. For example, a recent study by Chen et al. (28) on Chinese medical professionals found that stressors such as promotion pressure and medical disputes were differentially associated with mental health outcomes in men and women. Future research should therefore investigate these gender-specific pathways to burnout in the radiology nursing population to inform more targeted interventions.

In conclusion, this study explores the associations among work stress, effort–reward imbalance, and burnout among radiology nurses, providing evidence that high stress and ERI are significantly associated with an increased risk of burnout. Hospital administrators must address this by improving work environments, optimizing staffing, and refining evaluation and promotion systems which may be associated with lower levels of stress and burnout. Nurses should strengthen their self-regulation and resilience to navigate occupational challenges. These measures can enhance the quality and safety of radiological nursing, foster harmonious patient–provider relationships, and increase health care standards. Future research should investigate additional factors influencing burnout to inform more targeted interventions.

## Data Availability

The original contributions presented in the study are included in the article/supplementary material, further inquiries can be directed to the corresponding authors.
